# Surgical Site Infections after Deep Brain Stimulation Surgery: Frequency, Characteristics and Management in a 10-Year Period

**DOI:** 10.1371/journal.pone.0105288

**Published:** 2014-08-14

**Authors:** Silje Bjerknes, Inger Marie Skogseid, Terje Sæhle, Espen Dietrichs, Mathias Toft

**Affiliations:** 1 Department of Neurology, Oslo University Hospital, Oslo, Norway; 2 Department of Neurosurgery, Oslo University Hospital, Oslo, Norway; 3 Faculty of Medicine, University of Oslo, Oslo, Norway; Georgia Institute of Technology, United States of America

## Abstract

**Background/Aims:**

Deep brain stimulation (DBS) implant infection is a feared complication, as it is difficult to manage and leads to increased patient morbidity. We wanted to assess the frequency and possible risk factors of DBS related infections at our centre. In the purpose of evaluating treatment options, we also analyzed treatment, and the clinical and microbiological characteristics of the infections.

**Methods:**

Electronic medical records of all patients undergoing DBS surgery at our centre, from 2001 through 2010, were retrospectively reviewed.

**Results:**

Of the 588 procedures performed 33 (5.6%) led to an infection. Some patients underwent several procedures, thus 32 out of totally 368 patients (8.7%), and 19 out of 285 patients (6.7%) who received primary lead implantation, developed an infection. Most infections (52%) developed within the first month and 79% within three months. In the majority of the infections (79%) hardware removal was performed. Staphylococcus aureus infections were the most frequent (36%), and more likely to have earlier onset, pus formation, a more aggressive development and lead to hardware removal. No risk factors were identified.

**Conclusions:**

Our results indicate that infections with more severe symptoms and growth of staphylococcus aureus should be treated with local hardware removal and antibiotic therapy. In other infections, an initial trial of antibiotic treatment could be considered. New knowledge about the microbiology of DBS related infections may lead to more effective antimicrobial treatment.

## Introduction

Deep brain stimulation (DBS) has become an important treatment option in movement disorders resistant to medical treatment. Well-established indications are Parkinson’s disease (PD), essential tremor (ET) and dystonia. Other neurological indications are under evaluation, such as cluster headache, Gilles de la Tourette syndrome, refractory pain and epilepsy, as well as some neuropsychiatric indications [1], [2], [3].

DBS is performed as an elective procedure, can be performed safely and is well tolerated by most. However it is not without risk of complications, and system infection is a known problem [4]. As with any therapy involving permanent implants, infectious complications are feared, as they are difficult to manage. Infections are usually found at the site of the internal pulse generator (IPG), at the connector site or on the scalp where the lead exits the brain. Intracerebral infections are rare [5].

The vast majority of DBS-related infections are associated with bacteria belonging to normal skin flora, with Staphylococcus aureus and Staphylococcus epidermidis being the most commonly isolated pathogens [6], [7], [8]. Previous studies have reported very different frequencies of infectious complications, varying from 0% to more than 15% of operated patients [7], [8], [9]. These numbers are difficult to interpret for several reasons, including the lack of consensus regarding definition and criteria of infection, varying follow-up time, few included patients, different operating techniques and varying peri- and postoperative use of prophylactic antibiotics. Some centers only include those patients whose infections need surgical intervention and exclude those with superficial infections that can be cured with conservative therapy alone [10]. Also, few of the available articles have analyzed risk factors and characteristics of infection. Furthermore, there is a lack of evidence and consensus on best management, and this seems to vary widely from center to center.

Here we report the findings of a retrospective study of all patients receiving DBS treatment in our hospital within a ten-year period from 2001. We have evaluated the frequency, clinical and microbiological characteristics and management of surgical site infections. We have also examined the relationship between infections and a number of possible risk factors.

## Methods

All patients undergoing DBS surgery between 2001 and 2010 at Oslo University Hospital were included in this retrospective study, with a minimum follow-up of 12 months. The electronic medical records were used for documentation of surgical site infections and their clinical and microbiological characteristics, management and risk factors. In Norway this study design does not require ethical approval, as it is a retrospective internal quality control study. Internal approval was obtained from the Privacy Protection Officer at our hospital (Personvernombudet). All of the authors had contact with the included patients and participated in varying degree in patient treatment. Data files are anonymized with a code, stored in a secure and separate area, accessible only to the corresponding author. The patients who provided photographs for this study have consented in the use of the images for this purpose. We have not obtained consent from the patients to use their records as this was set up as an internal quality control study, and thus this is not required.

### Surgical procedures

DBS surgery was performed as described previously [11], [12]. In brief, MRI was performed the day before surgery. On the morning of the surgery the CRW stereotactic frame was mounted under local anesthesia before stereotactic 3D CT imaging was performed and fused with the MRI, and trajectories and targets were then planned. Intraoperatively the target of the permanently implanted electrode was further refined by a combination of microelectrode recordings and intraoperative test stimulation. Electrodes were then fixed to the skull, extension leads connected, and a Kinetra, Soletra or Activa (Medtronic, MN, USA) neurostimulator implanted under general anesthesia.

Implantation of a new DBS system was always performed as the first surgery of the day, using standard operating room technique. The preparation of the patients for surgery, which included full shaving of the scalp, cleaning with chlorhexidine 5 mg/ml and draping with Ioban drape over the complete surgical area, was performed by the surgical nurse. Surgeons used double layer gloves, and the number of people in the operating room was limited. Intravenous Cefalotin 2 grams was administered just before the start of the surgery, and was followed by 1 gram every 3 hours perioperatively. In case of possible allergy to Cefalotin, clindamycin, or in a few cases erythromycin or cloxacillin, were used. All procedures were performed during the same operation, also when electrodes were implanted bilaterally. The stimulator was turned on the first or second postoperative day, and most patients were admitted for 7–10 days before being discharged to their home.

Replacement of the IPG due to depleted battery was usually performed under local anesthesia, although if needed it was occasionally performed under general anesthesia. This procedure was usually not performed as the first surgery of the day. The subcutaneous pocket was opened via the old scar, before the old IPG was disconnected and replaced by a new generator. Prophylactic antibiotics were administered just before the surgery. The surgeries were most often performed as day surgery, but in some cases the patients spent a day or two if they needed to recover and to adjust the stimulator parameters. We have not routinely used any local antibiotic irrigation.

### Infections

**Figure 1:**
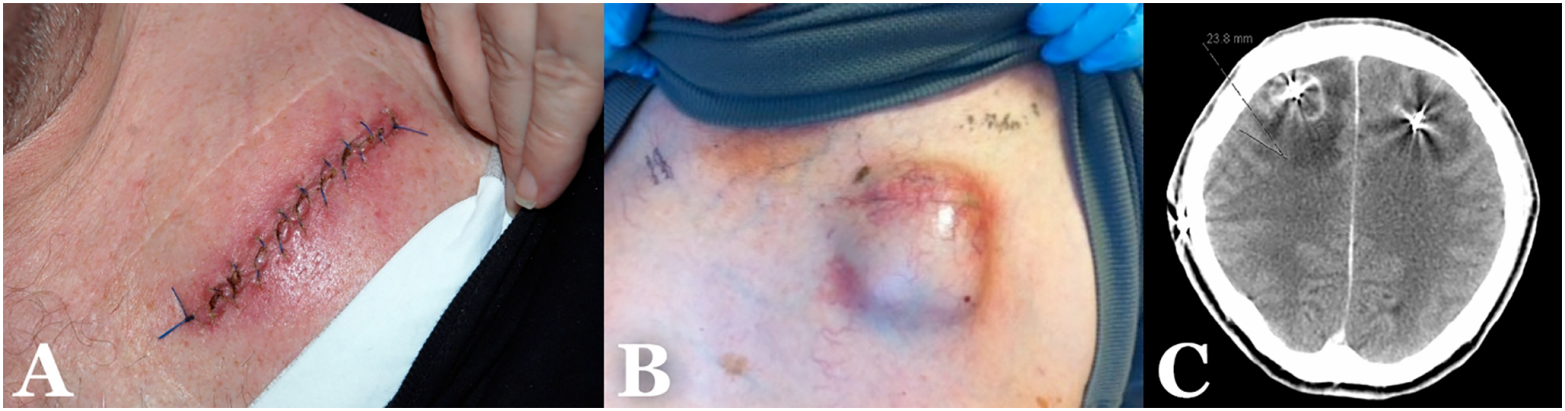
Examples on the different types of infection. A) Superficial incisional SSI. B) Deep incisional SSI. C) Organ/space SSI. (SSI = surgical site infection).

For definition of infection we used criteria provided by the Guideline for Prevention of Surgical Site Infection (SSI), which distinguishes between superficial incisional SSI, deep incisional SSI and organ/space SSI [13]. According to the criteria, superficial SSI involves only skin and subcutaneous tissue of the incision and has to occur within 30 days of the operation. Deep SSI involves deep soft tissues and organ/space SSI involves any part of the anatomy which was opened or manipulated during the operation. Both deep incisional SSI and organ/space SSI had to occur within one year of the operation if an implant was left in place (Figure 1A–C). To diagnose an infection at least one of the following was required: 1) purulent drainage, 2) organisms isolated from aseptically obtained culture, 3) at least one of the following sign or symptoms of infection: pain or tenderness, localized swelling, redness, heat, fever (>38C) or spontaneous dehiscence, 4) an abscess or other evidence of infection found on direct examination, during reoperation or by histopathologic or radiologic examination, 5) diagnosis of an SSI by a surgeon or attending physician [13].

### Statistical analyses

Descriptive statistics were calculated for baseline demographic and clinical data. Possible risk factors for infection, such as age, smoking, diabetes mellitus, diagnosis, DBS target, date of surgery (month and year) were first analyzed by group comparisons using chi-square tests for categorical data and t-tests or Mann-Whitney U test for continuous data. Regression analysis was then performed for possible risk factors. P<0.05 was considered statistically significant. All statistical analyses were performed using SPSS version 18.0.

## Results

### Study population

During the ten-year period from 2001 through 2010, a total of 588 surgical procedures were performed in 368 patients (145 women/223 men). This includes initial implantations (n = 286, of these 22 with unilateral lead), IPG replacements (n = 280) or replacement after successfully treated infections (n = 22, one with unilateral lead). The total number of operations has steadily increased in the study period. Numbers of initial implantations have been quite stable, but there has been an increase in IPG replacements, especially in 2010.

Mean age at surgery was 60 years (range 3–84 years). 59 patients (16%) were smokers, and only three patients had diabetes mellitus. The most frequent diagnosis was PD with 270 patients (73%), tremor disorders follow with 60 patients (16%) and primary dystonia with 33 patients (9%). Our study group also included three patients treated for secondary dystonia; two with PKAN (pantothenate kinase-associated neurodegeneration) and one with multiple sclerosis (MS). Another two patients were treated for tremor, due to atypical Parkinsonism (initially misdiagnosed as PD) and MS respectively. Considering target, the most common was nucleus subthalamicus (STN) in 225 patients (61%), followed by the ventral intermediate nucleus of thalamus (ViM) in 105 patients (29%) and internal globus pallidus (GPi) in 38 patients (10%).

### Infections

In our analyses we included 32 patients with 33 infections. We classified 8 as superficial incisional SSI and 23 as deep incisional SSI. An additional two was classified as organ/space SSI, where one presented with headache and brain abscess, and the other with fever, elevated CRP and meningitis due to penetration through the skin of the scalp. Both recovered fully without any neurological deficit. The most typical location of infection was over the IPG box (28/33). Table 1 shows data on all infections.

**Table 1:** Data on all the cases of infection.

Age/sex	Diagnosis/lesion	Surgicalprocedure	Time of infect.afterprocedure	Focus ofinfection	Superficialvs Deep	Purulentdrainage	Culture	Trial ofconserv. treatm.	Woundrev.	Removal ofhardware
M/51	PD/STN	Implantation	0	IPG, cable and electrodes	organ/space	yes	S. aureus	yes	-	IPG + cable/electrode B
M/10	PKAN/GPi	Implantation	7	IPG	deep	yes	S. aureus	-	-	IPG
M/52	ET/ViM	IPG replacem	7	IPG + cable+ meningitis	organ/space	yes	S. aureus	-	-	IPG + cable/electrode B
M/73	PD/ViM	IPG replacem	12	IPG	deep	yes	S. aureus(also in bl.culture)	yes (only 1day)	-	IPG
F/11	PKAN/GPi	Implantation	14	IPG	deep	yes	S. aureus	-	-	IPG
F/70	PD/STN	Implantation	16	IPG	superficial	yes	S. aureus	yes	-	-
F/72	PD/ViM	IPG replacem	28	IPG	deep	yes	S. aureus	-	-	IPG
F/66	PD/STN	Implantation	35	IPG + postaur.	deep	yes	S. aureus	yes	-	All
M/65	PD/STN	Implantation	35	Frontal R	deep	yes	S. aureus	yes	-	Electrode R
M/42	PD/STN	Implantation	38	IPG + postaur.	deep	yes	S. aureus	yes	-	IPG + cable
M/65	PD/STN	Reimpl.	75	IPG + postaur.+ Frontal R	deep	yes	S. aureus	yes	-	IPG/cable/electrodes B
F/61	PD/STN	Implantation	212	IPG + cable	deep	yes	S. aureus	yes	-	IPG + cable
M/50	PD/STN	IPG replacem	14	IPG	superficial	-	CoNS	yes	yes	-
F/61	PD/STN	Implantation	28	Frontal R	superficial	yes	CoNS	yes	yes	-
M/61	ET/ViM unilat.	IPG replacem	70	IPG	deep	yes	CoNS	-	-	IPG
F/75	PD/ViM	IPG replacem	84	IPG	deep	-	CoNS	-	-	IPG/cable
M/56	ET/ViM	IPG replacem	87	IPG	deep	yes	CoNS	-	-	IPG
F/67	PD/STN	IPG replacem	182	IPG	deep	-	CoNS	-	-	IPG
F/62	PD/STN	Implantation	365	IPG	deep	-	CoNS	-	-	IPG
F/35	Dy/GPi	Implantation	365	IPG + postaur.	deep	-	CoNS	-	-	Cable
F/67	PD/ViM	IPG replacem	1	IPG + postaur.	deep	yes	Skin flora	-	-	IPG + cable
F/62	PD/STN	IPG replacem	14	IPG	superficial	-	Skin flora	-	yes	IPG
M/65	PD/STN	IPG replacem	21	IPG	superficial	-	Skin flora	-	yes	IPG/cable
M/77	PD/ViM	IPG replacem	112	IPG	deep	-	Skin flora	-	-	IPG
F/30	Dy/GPi	Implantation	0	IPG	deep	-	Negative	-	yes	IPG
F/72	PD/ViM	Implantation	2	Frontal R	deep	-	Negative	yes	-	-
M/70	ET/ViM	Implantation	9	Frontal L	superficial	-	Negative	yes	-	-
M/55	PD/STN	Implantation	14	Frontal L	superficial	-	Negative	yes	-	-
M/62	PD/STN	Implantation	42	IPG	deep	-	Negative	yes	-	IPG/cable
M/67	PD/STN	Implantation	90	IPG	deep	-	Negative	-	-	IPG/cable
F/71	PD/STN	Implantation	180	IPG	deep	yes	Negative	-	-	IPG/cable
F/72	PD/GPi	IPG replacem	273	IPG	deep	yes	Negative	-	-	IPG
M/61	PD/STN	Implantation	14	IPG	superficial	-	NA	yes	-	-

(M = male, F = female, PD = Parkinson’s disease, Dy = dystonia (primary), ET = essential tremor, PKAN = pantothenate kinase-associated neurodegeneration, STN = nucleus subthalamicus, ViM = ventral intermediate nucleus of thalamus, GPi = internal globus pallidus, IPG = Implanted Pulse Generator, replacem = replacement, reimpl = reimplantation, postaur = postauricular, R = right, L = left, S. aureus = Staphylococcus aureus, CoNS = coagulase-negative Staphylococci (includes S. epidermidis and S. capitis), NA = not applicable (test not obtained), B = bilateral).

Of the 588 procedures performed, 33 (5.6%) was complicated by an infection. In total over the ten year study period, 32 of 368 patients (8.7%) were treated for infection. Seventeen of the infections (52%) developed within the first month, and 26 (79%) within the first three months. During our study period two patients developed infections that occurred later than 1 year after surgery. Thus they do not fulfill the criteria for SSIs and were not included in our study. These infections developed in the second year after surgery and were both due to chronic erosion of the skin. The frequency of infections did not differ significantly between primary implantations (6.7%) and subsequent procedures (4.6%) (table 2). Neither did it differ between the three main diagnostic groups (PD, ET and dystonia) or the different targets. None of the patient related factors (gender, age at surgery, diabetes mellitus or smoking status) where associated with an increased risk of infection (table 3). However, of the 36 patients treated for dystonia (33 with primary dystonia, 2 with PKAN and one with MS) all the four patients who contracted an infection suffered from generalized dystonia (two with primary generalized dystonia and both of the PKAN patients), which in three of them had led to a low body mass index at the time of surgery.

**Table 2:** Number of infections in relation to type of procedure.

	Number of procedures	Number of infections	Frequency (%)
**Implantations**	286	19	6.6%
**IPG replacements**	280	13	4.6%
**Reimplantations**	22	1	4.5%
**Total**	588	33	5.6%

Most of the infections appeared after initial implantations, but there are no significant differences between the groups. Comparing all three; p-value = 0.57. Comparing only implantation and IPG replacements; p-value = 0.30. Both with chi-square test. (IPG = internal pulse generator).

**Table 3:** Number of infections in relation to possible risk factors.

	Non-infection	Infection	p-value
**Diabetes Mellitus**	3	0	1.00 (Fishers exact test)
**Smoking** *	54 (16.1%)	5 (15.2%)	0.77 (chi-square)
**Age (mean)**	60.0	58.4	0.52 (t-test)
**Sex (M/F)**	206/129	17/16	0.26 (chi-square)

(M = male, F = female).

*21 with unknown smoking status.

**Figure 2:**
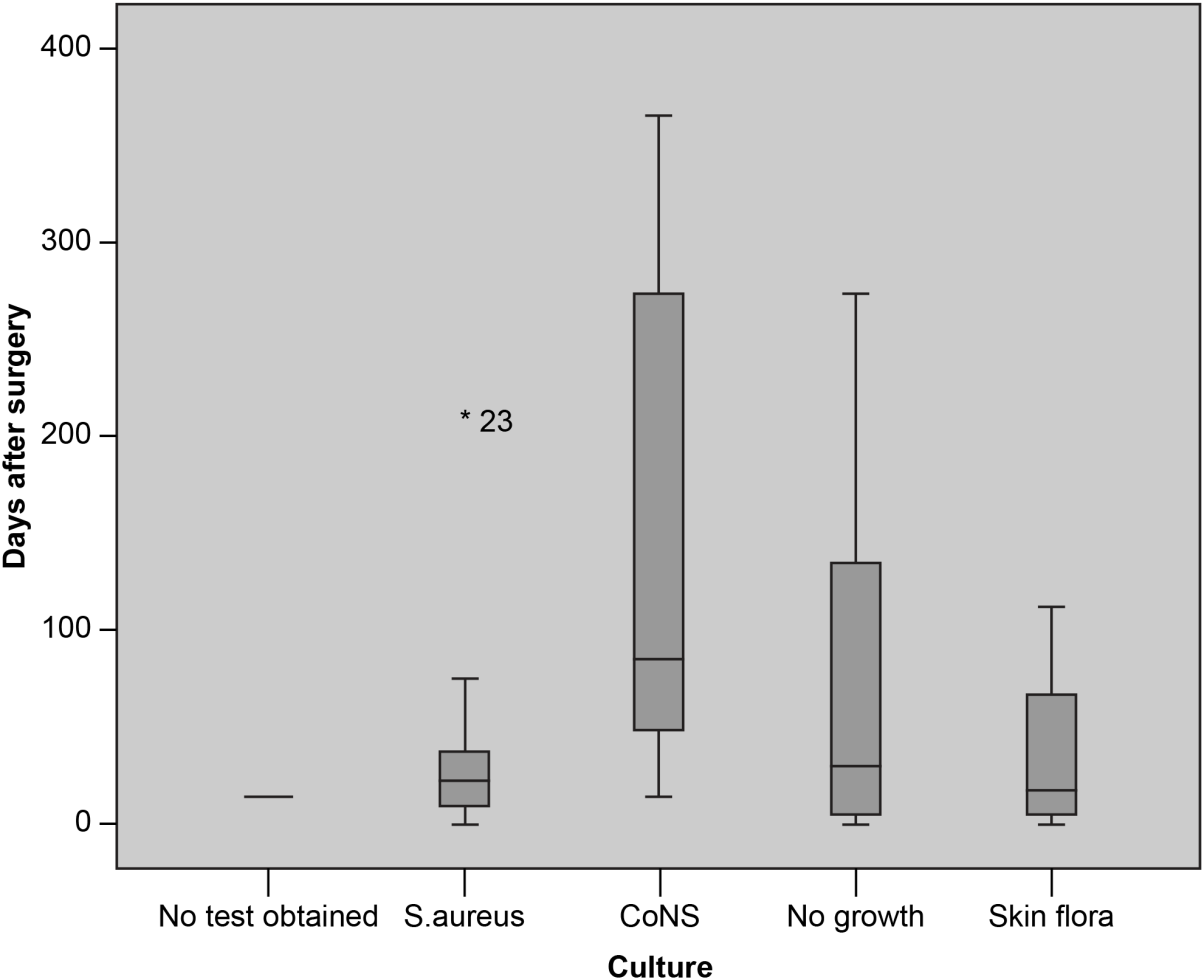
Number of days until onset of infection compared with culture results. S. aureus infections have earlier onset than in the CoNS group (p-value 0.02, Mann-Whitney U Test). (S. Aureus = Stahylococcus Aureus, CoNS = coagulase negative staphylococci).

Bacteriological tests were obtained in 32/33 cases of infection. They showed growth of S. aureus in 12 patients (36%), coagulase-negative Staphylococci (CoNS; includes S. epidermidis and S. capitis) in 8 (24%) and normal skin flora in 4 (12%). Eight patients had negative cultures (24%). None of the identified S. aureus strains were methicillin resistant. S. aureus infections had earlier onset than infections caused by CoNS (figure 2). All of the S. aureus positive infections (12/12) had purulent drainage, and 10/12 was classified as deep SSIs. Only four patients had a markedly increased level of c-reactive protein (CRP 90–>200), all of these had growth of S. aureus in wound culture. Two patients had a less pronounced elevation of CRP (11 and 36), one with S. aureus and one without growth.

### Treatment

**Figure 3:**
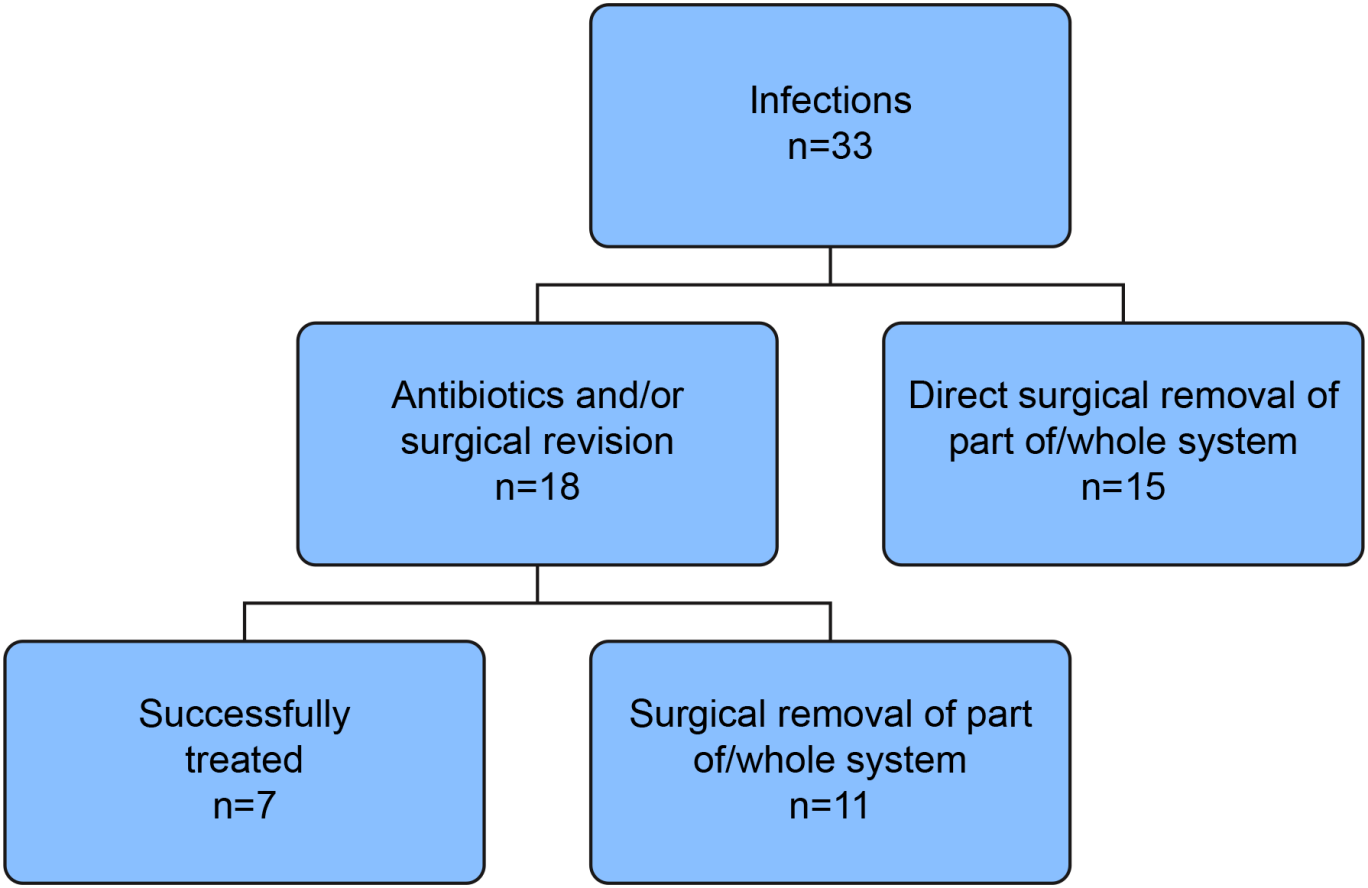
Infection treatment flowchart.

At our center there has been a policy to try a conservative approach in surgical site infections related to DBS, in order to avoid unnecessary removal of the hardware. We had no established guidelines for immediate explant. The treating physicians took the decision together, after considering the seriousness of the infection. Our results indicate that, for uncertain reasons, the earlier the infection debut, the higher the likelihood that a conservative approach was tried first. Mean days until debut of infection was 124.3 for patients explanted immediately, whereas patients initially treated with antibiotics or surgical revision first had mean debut of infection 32.3 days after surgery. We tried treatment with antibiotics and/or wound revision in 18 of the 33 cases of infection, unfortunately it was only successful in seven of them. In the remaining 11 patients, it became necessary to remove part of, or in a few cases the whole system (see figure 3). Seven of these (64%) were positive for S. aureus in culture, compared to only one (14%) of the seven who were successfully treated by the conservative approach. Whereas nearly all of the deep SSIs (22/23) and both the organ/space SSIs (2/2) were treated with hardware removal, six of the eight superficial SSIs were treated conservatively (p<0.001). The presence of purulent drainage had little impact on the decision to remove any part of the system, as the majority in both groups went through hard-ware removal (16/18 patients with purulent infections and 10/15 with non-purulent infections, p-value 0.26). Thus, 26/33 infections ended up with partial or total removal of the system. However, in 21% of the patients with surgical site infections the hard-ware could be saved. The removal of the infected part of the system, together with antibiotic treatment, always resulted in clearing of the infection, without neurological sequelae.

During the ten-year period that we report from, the antibacterial treatment strategies to treat implant infections have varied at our hospital. This is reflected in the large variability of the antibacterial treatment regimens used. Treatment duration was most often 2–6 weeks (range two days to thirteen weeks). So many different combinations of antibiotic treatment were used, and with variable duration, that it was impossible to draw any conclusions from our data about which regimen may be more efficient.

## Discussion

The main strength of our study is the large number of patients and the long follow-up period of at least one year post surgery. Also, we expect to have a complete set of data, as we are the only center in Southern Norway performing DBS surgery. We follow up all our patients at regular intervals as both in- and out-patients, and have a specialist nurse they can call during business hours if any problems arise. Thus, we are confident that we would be notified if our patients would be treated for surgical site infections at other institutions.

The frequency of infections (5.6% of performed procedures) in our study is in the same range as described in previous DBS studies. However there is a wide variability in the reported frequency of infections in the literature, and this can have a multitude of reasons. In some studies the infection rate is calculated by the number of patients only, while others have calculated by number of implants or procedures. Studies use different inclusion criteria, some have included all infections whereas some only included surgically treated infections. In addition, the follow-up time is very variable.

### Risk factors

We could not find any significant differences in infection rate when comparing type of surgery (initial versus IPG change), main diagnostic groups (PD, ET, dystonia), targets (STN, ViM, GPi) or sex, age, diabetes mellitus or smoking status. However it should be noted that the numbers are small so that caution is required when interpreting the statistical results. Also other reports have failed to show a statistically significant association with DM, smoking and sex [14], [15]. This is unlike the findings in a recent article by Pepper et al where the infection rate was higher in the replacement surgery group and for male patients [16]. Also Bhatia et al found a higher incidence of infection in the replacement group [17]. But the numbers of patients included in these studies are even smaller. Pepper et al proposes that a reason could be the timing of surgery during the day, but in both our centers the primary implantation is performed as first of the day and the IPG replacements follows later in the day. They also propose that the fibrous pocket around IPG do not provide an adequate inflammatory response as during the primary procedure, but this should then also be true for our study.

### Clinical microbiology

As in our study, the most frequent microorganisms found in culture in other studies of DBS infections and cardiovascular implantable electronic device infections are S. aureus and CoNS [6], [8], [18], [19]. In our study we found no gram negative infections, even though recent findings from the United States shows that of healthcare-associated infections and neurological SSIs, gram negative agents account for 29% and 21% respectively [20]. In accordance with the same study the two most frequent pathogens were S. aureus and CoNS. Also, we note that we did not find any MRSA positive infections, reflecting the generally low frequency in Norway [21].

Just above 50% of the infections in our study occurred within the first month post surgery and 79% within the first three months, median 28 days. Several other studies report similar numbers, but a study from 2010 found that most of their infections occurred later than 30 days, and only 7/33 (21%) during the first month after surgery (median 64 days) [8].

Infections caused by S. aureus started significantly earlier, had more often purulence and other classical signs of infection, and were less successfully treated with antibiotics and/or wound revision alone. Markedly increased CRP was seen exclusively in S. aureus infections, but in a minority even in these patients. Also, it is not an uncommon finding on the second or third postoperative day after DBS surgery in patients without infection. Thus CRP is a poor marker for surgical site infections. We also found that S. aureus infections accounted for nearly all of the cases in which the infection spread along the system with several foci. This concurs with a study on the role of biofilm in staphylococcal infections of ventricular assist device driveline infections, where S. aureus exhibited a more rapid migration than S. epidermidis [22].

### Treatment

In our series of surgical site infections, initial antibiotic therapy and/or wound revision alone was successful in 7/33 (21%), in which removal of hard-ware then could be avoided. Hard-ware were partially saved in 22/33 (67%), but complete removal was performed in 4/33 (12%). However in the study by Bhatia et al. they were able to preserve part of or the whole hardware only in about 50% of the patients. In their study they had a higher proportion of infections localized over the burr holes [8]. Gorgulho et al reported that 12/20 cases of infection underwent complete hardware removal, but they had only attempted a conservative approach when symptoms were less pronounced [6]. Many studies do not specify if whole or part of the system was removed, and some only report it per patient and not per procedure/infection. Our results indicate that partial removal of hardware together with antibiotics should be attempted if the infected part is the IPG or extension wire.

Of the seven patients treated successfully conservatively, two had revision in combination with varying forms of antibiotics for about 4 weeks (clindamycin + cefotaxime or cloxacillin + cefuroxime + linezolid). The remaining five patients all received dicloxacillin in monotherapy, from 1 week–13 weeks (mean 4.4 weeks). Of the conservatively treated patients six were classified as superficial SSIs and only one was caused by S. aureus. Since the antibiotic regimens have varied considerably throughout the ten-year period of this study, we have not been able to draw any further conclusions about the effectiveness of the different regimens. Because the most frequent microbiological agents causing surgical site infections are S aureus and CoNS, the antibacterial therapy should include coverage for these while awaiting culture results.

### Diagnosis

In some cases the diagnosis of infection was not clear in the beginning, with minimal and indolent symptoms. Some authors define erosions as non-infections, whereas others view them as likely manifestations of an underlying infection with a less virulent organism. In our study most patients exhibited typical local symptoms such as erythema, swelling, pain and pus formation, most commonly localized around the IPG. But we also included several patients with just erosion, discoloration and/or non-purulent secretion. It could be that the wounds are in different stages of the infection spectrum, or possibly that the pathogen produces different clinical pictures. It is known that the infection detection rate is dependent on the type of criteria used, and that the detection rate is lower when pus is used as only criterion. For this reason Cutting and Harding put up a list of criteria for identifying wound infection in 1994, reviewed by Cutting and White in 2004 to define criteria that are indicative of infection in different wound types [23], [24]. These criteria may be used in addition to the CDC criteria to diagnose surgical site infections, especially when the clinicians are in doubt. Since different centers around the world use different criteria for infection, specific and validated criteria for DBS infections seems to be needed to aid clinicians to diagnose and treat patients correctly.

### Biofilm

Recent research have shown that the staphylococcal species, notably S. aureus and CoNS, have the ability to form surface-associated biofilms on implants, that make them highly resistant to host defense mechanisms and antibiotics, and thus promotes persistent infection [25], [26]. The strains of staphylococci that grow in biofilm, Small Colony Variants (SCV) and so-called “persister” cells, are difficult to grow in conventional culture and are only found if extended culture or specific identification techniques are applied. They can be misdiagnosed by microbiologists because they are easily overgrown and missed when the normal staphylococci are present [27]. This may be the reason why there are relatively few descriptions of such infections in the literature and may explain why negative cultures may occur, even when there is obvious pus formation. This may be the case in some of the patients in our series with negative culture, as extended cultures were not routinely used at our hospital. Biofilm formation may also be an explanation for the failure of response from traditional antibiotic therapy that was given as first line treatment in many of our patients. In the future, we should bear in mind the possibility of biofilm formation and search for these staphylococcal variants. A promising and recommended therapy for this is co-administration of rifampicin together with other antibiotics (β-lactam antibiotics or vancomycin), which has been consistently successful in clearing biofilm infections in vitro, in vivo and in clinical studies [28], [29].

### Nasal carriers of S. aureus

It is our goal to avoid the removal of any part of the DBS system. The cessation of electrical stimulation is troublesome to the patient, who will experience severe worsening of disease symptoms. We also aim to maintain the intracerebral lead(s) in all scenarios, as lead replacement is very time consuming and resource demanding. It is known that nasal carriers of S. aureus are at increased risk for health care-associated infections with this organism. Decolonization has been shown to be effective in reducing the risk, especially for deep surgical-site infections [30]. In our unit this test has only been performed since 2008, and even after this the usage has been variable. This could be a point of improvement in our routines, as a rapid screening and decolonization on admission could possibly reduce the infection risk.

### Conclusions

It could be expected that there will be an increase in the number of DBS related infections in the future, as the procedure becomes more and more prevalent, both for movement disorders and other indications. A clear definition of device infection and guidelines for treatment are needed. Our results indicate that when infection caused by S. aureus is suspected, the therapy should be prompt local hardware removal and long term antibiotic therapy. In other infections an initial trial of antibiotic treatment should be considered. The awareness of biofilm formation and the treatment for this requires further attention and research, as this could potentially lead to higher success in treating the patients in a conservative manner and lead to less morbidity and healthcare costs related to the management of DBS infections in the future.
